# Factors influencing the determination of cell traction forces

**DOI:** 10.1371/journal.pone.0172927

**Published:** 2017-02-24

**Authors:** Manuel Zündel, Alexander E. Ehret, Edoardo Mazza

**Affiliations:** 1 Institute for Mechanical Systems, ETH Zurich, Zurich, Switzerland; 2 Empa, Swiss Federal Laboratories for Materials Science and Technology, Dübendorf, Switzerland; LAAS-CNRS, FRANCE

## Abstract

Methods summarized by the term Traction Force Microscopy are widely used to quantify cellular forces in mechanobiological studies. These methods are inverse, in the sense that forces must be determined such that they comply with a measured displacement field. This study investigates how several experimental and analytical factors, originating in the realization of the experiments and the procedures for the analysis, affect the determined traction forces. The present results demonstrate that even for very high resolution measurements free of noise, traction forces can be significantly underestimated, while traction peaks are typically overestimated by 50% or more, even in the noise free case. Compared to this errors, which are inherent to the nature of the mechanical problem and its discretization, the effect of ignoring the out-of-plane displacement component, the interpolation scheme used between the discrete measurement points and the disregard of the geometrical non-linearities when using a nearly linear substrate material are less consequential. Nevertheless, a nonlinear elastic substrate, with strain-stiffening response and some degree of compressibility, can substantially improve the robustness of the reconstruction of traction forces over a wide range of magnitudes. This poses the need for a correct mechanical representation of these non-linearities during the traction reconstruction and a correct mechanical characterization of the substrate itself, especially for the large strain shear domain which is shown to plays a major role in the deformations induced by cells.

## Introduction

The mechanical interaction of cells with their surrounding has been shown to be a key component in various cellular processes, such as cell proliferation, differentiation and migration [1, 2]. The quantitative analysis of this interaction is relevant for investigations in mechanobiology and a broad palette of corresponding methods has been developed during the last decades, gathered under the term Traction Force Microscopy (TFM) methods [3, 4]. Most of these methods share the same fundamental idea: microscope imaging is employed to visualize the displacement field induced by the cell on a substrate with known mechanical properties and this information is successively fed into a mechanical model of the system, allowing to solve the inverse, ill-posed problem and to retrieve the traction forces originally applied by the cell. TFM has nowadays become a versatile tool for studying cellular forces in various fields, e.g. durotaxis [5], collective cell motility [6] and cancer research [7].

The two most prominent TFM platform types are based on arrays of elastic micro-posts (or pillars) [8, 9] or exploit flat continuous, soft and highly deformable substrates [10–12]. While pillared substrates lead a to simple mechanical system for which the forces applied by the cells to the pillars through their Focal Adhesions (FAs) can be computed using beam theory, the topology of this substrates has been shown to influence the behaviour of the cell [13, 14] and could therefore potentially bias the results. This issue, together with the significantly more complicated substrate manufacturing, has led to the broad acceptance of TFM platforms based on continuous flat substrates with known mechanical properties. For these methods, the measurement of the forces exerted by the cell on the substrate surface can be subdivided into two main steps: *i)* The determination of the displacement field induced by the cell on the substrate surface, often measured with the help of fluorescent markers and image processing methods and *ii)* the subsequent computation of cellular traction forces based on the solution of the inverse mechanical problem. The latter requires a suitable material model describing the substrate behaviour, and an appropriate numerical framework.

**Fig 1:**
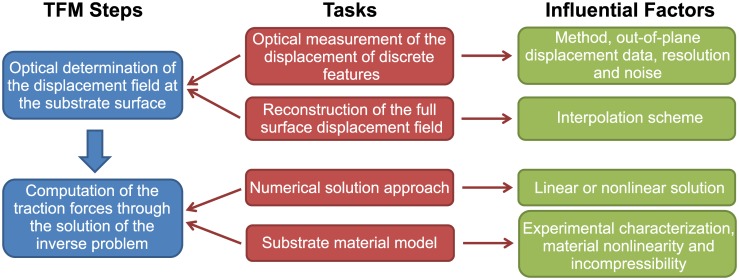
Main steps in TFM analyses and associated influential factors.

As delineated in Fig 1, both steps consist of different tasks, which are associated with factors that potentially influence the final results of the traction force reconstruction. Due to the many applications of TFM, the effect of these factors, both on the experimental setup and the solution of the inverse problem have been investigated in several works. Sabass et al. analysed the performance of Fourier Transform Traction Cytometry (FTTC) and Boundary Element Method (BEM) [15], two different numerical methods for solving the linearised inverse TFM problem based on the Boussinesq-Green function [16] for 2D traction fields. An extension of this solution approach to 3D traction fields has recently been proposed by del Álamo et al. and employed to investigate the difference between 2D and 3D TFM reconstruction, as well as the influence of substrate thickness [17]. Hur et al. evaluated the influence of measurement noise on the reconstructed traction stresses for linear Finite Element Analysis (FEA) based TFM method [18]. While these studies were based on the linearised solution, soft substrates employed in TFM can undergo deformations beyond the linear elastic regime, thus requiring a large strain TFM approach [4]. The comparison of small to large strain implementations of the TFM approach reported by Toyjanova et al. highlights the need for the latter, as the error in the traction stresses due to the linearisation exceeds 30% for moderate cell induced substrate deformations (strains in the order of 50%) [19]. Errors in a similar range were reported by Boudou et al. for TFM on polyacrylamide gels when neglecting the nonlinear mechanical behaviour of the substrate material [20].

The present study provides a systematic analysis and quantitative comparison of the influence of all factors indicated in Fig 1, using the same TFM platform and taking into account the non-linear characteristics of the problem. In particular, we quantify the influence of the displacement measurement quality (resolution and noise) as well as on the impact of different interpolation strategies for the reconstruction of the substrate displacement field, starting from the displacement of the measured discrete optical features. Our analysis is independent of the optical method applied to quantify of the substrate displacements such as the often used Particle Image Velocimetry or the more advanced Free Form Deformation algorithm [21].We also do not consider the influence of different regularization schemes used to reduce the effect of measurement noise, which are widely discussed in literature [15, 22–24]. Focusing on TFM for flat, continuous and highly deformable substrates, we will assess the importance of an appropriate substrate material characterization required to parametrize constitutive equations and the necessity for performing a non-linear mechanical analysis to compute cellular traction forces. The results identify critical aspects of the procedure for the reconstruction of traction forces, which need to be accounted for in order to avoid significant errors.

## Methods

### In-silico generation of TFM input data

**Fig 2:**
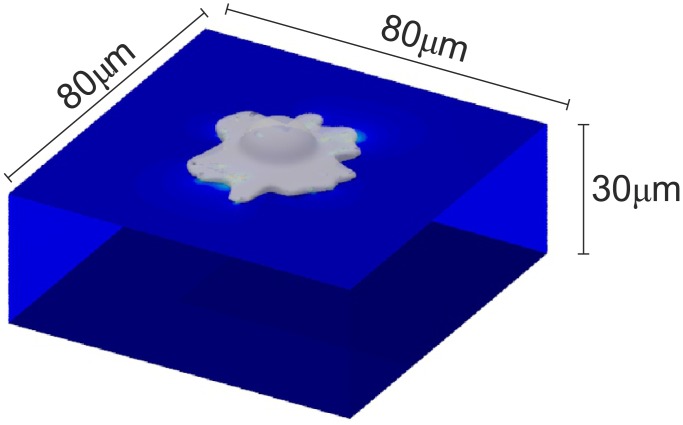
Visualization of the in-silico testcase: A cell deforming a substrate with finite size.

**Fig 3:**
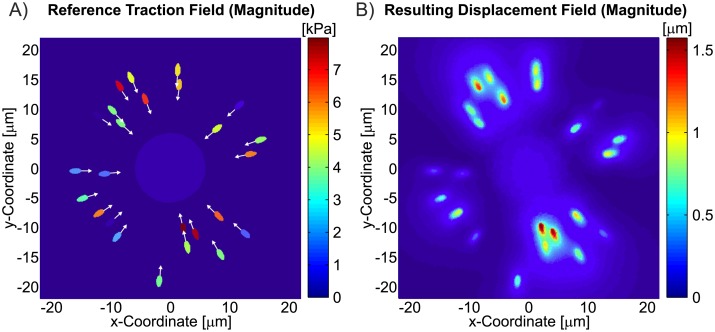
A) Reference traction field applied to the substrate, simulating a cell with 25 focal adhesions. The white arrows indicate the in-plane direction of the resulting traction force of each FA. B) Displacement field of the substrate surface resulting from the applied tractions. Both fields are plotted in the deformed configuration for material A and *ν* = 0.499.

For the investigations presented in this work, the input data needed for TFM was generated in-silico, mimicking a cell acting on a soft hyperelastic planar substrate with finite size (Fig 2) and a Young’s modulus of approximately 10kPa. For simplicity, it was assumed that the cell is only applying tractions trough reasonably sized FAs, neglecting other types of interaction such as podosomes and very small adhesion areas [25, 26]. A total of 25 FAs were modeled as ellipses (2*μm* x 1*μm*) randomly distributed around, and pulling towards the cell center with homogeneous traction stress magnitudes up to 8kPa, equivalent to a maximum of 12nN per FA (Fig 3A). These values were chosen based on data reported in literature for different cells analysed with TFM on substrates with similar stiffness [27–30]. To emulate the out-of-plane tractions that cells exert on flat substrates [31–33], each traction vector was inclined by an angle of 10° with respect to the surface. To equilibrate those traction components, the cell was assumed to push the substrate downwards in the center region. This effect was modelled with a traction stress acting in negative *z* direction applied to a circular area with 6*μm* radius (Fig 3A).

**Fig 4:**
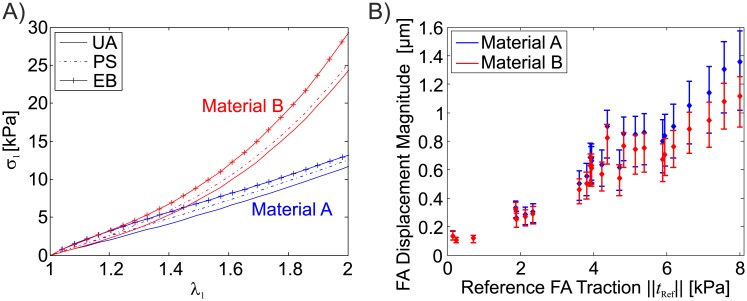
A) Stress-Stretch response for uniaxial tension (UA), pure shear (PS) and equibiaxial tension (EB) deformation modes for neo-Hookean (Material A, blue) and Ogden (Material B, red) materials for *ν* = 0.499. B) Displacement magnitude within each FA measured in the in-silico test-case plotted against traction. Reported are means (diamond shaped marker) with minima and maxima (errorbar) of each FA for the computations with both materials A (blue) and B (red).

The resulting traction force field (reference traction field) was applied on the surface of a rectangular section of the substrate (80*μm* x 80*μm*, thickness: 30*μm*), modelled with tetrahedral finite elements in Abaqus 6.10EF. While all displacement degrees of freedom were fixed on the bottom face of the substrate, the lateral faces were constrained by symmetry boundary conditions, restraining the displacement of those nodes within their original vertical planes. The resulting displacement field (Fig 3B) was computed using two different hyperelastic substrate materials both characterized by a shear modulus of 3.33 kPa: a neo-Hookean material (A), and a 2-term Ogden material (B) (Fig 4A), with pronounced non-linear stress-strain characteristics. The effect of the material law on the displacement magnitudes within each FA in the in-silico test case is shown in Fig 4B, which correlates FA displacement and traction force magnitudes. Note that the reported FA displacements converge to a finite value for low tractions since weak FAs are dragged along by stronger neighbours. To investigate the influence of the volumetric behaviour, different values of Poisson’s ratio were used (*ν* = 0.45, *ν* = 0.499). Except for the investigations presented in section *‘Modelling of geometric and material nonlinearities’*, it was assumed that the substrate material used in the in-silico computation is known, so that the mechanical behaviour of the substrate is captured exactly during the reconstruction.

**Fig 5:**
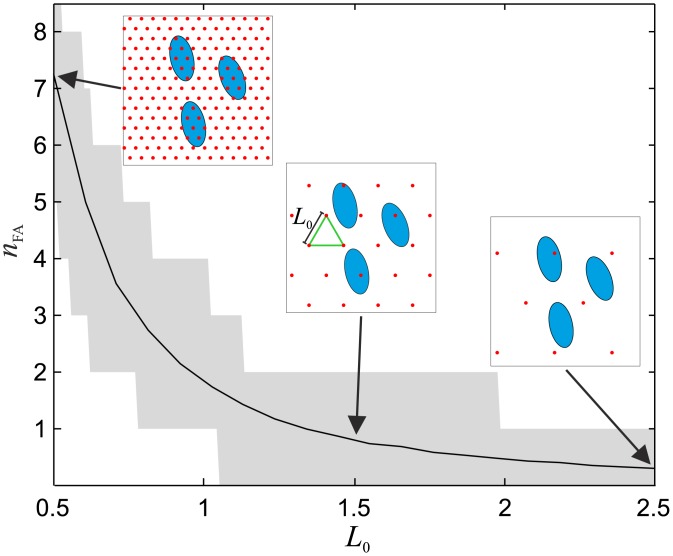
The relation between fluorescent markers distance (*L*_0_) and the quantity of fluorescent markers that are positioned within a focal adhesion (*n*_*FA*_). Reported results have been evaluated numerically for 25000 elliptical focal adhesions (2*μm* x 1*μm*). The black solid line marks the average, the area shaded in grey the maximum and minimum.

Due to the extremely high mesh density of the finite element model (100nm element size around the FAs) and the corresponding numerical resolution, the computed displacement field (Fig 3B) and its corresponding traction stress field are interpreted as a *virtual ground truth*, i.e an exact reference case not affected by any error. The in-silico generated displacement fields are used to extract the displacement vectors at the discrete locations of *virtual fluorescent markers*, as it would be the case in a real TFM experiment. For simplicity, these markers are distributed on a regularly spaced triangular array with marker distance *L*_0_ (in reference configuration), as actually realized in [30] using nanodrip printing technology to print the marker array directly on a silicone-based TFM substrate [34]. *L*_0_ is therefore coupled to the area density of the fluorescent markers on the substrate surface, defining the measurement resolution of the displacement field during TFM. In order to account for the randomness of the relative position of the marker array with respect to the FAs, the displacement vectors were extracted multiple times for the same *L*_0_ (5–25 testcase repetitions), shifting randomly the triangular grid, allowing a robust evaluation of the reconstruction quality. If not specifically noted otherwise, the results presented in this study are obtained for displacement field measurements with *L*_0_ = 1*μm*. The relation between *L*_0_ and the average number of fluorescent markers that are positioned within a focal adhesion is reported in Fig 5.

Note that the present investigation assumes that the field of view is large enough to visualize the cell in its full extent and extract the displacement field under and near the cell. In cases where cells are very close to the boundaries of the field of view (or partially cross these boundaries), traction force determination is affected by the interaction with the systems boundaries, thus leading to additional reconstruction errors.

Contrary to our in-silico case, experimentally measured marker displacements are affected by noise. To investigate potential consequences, random noise was added to the virtually measured displacement vectors prior to the traction reconstruction in our study for the results presented in section *‘Displacement field noise’*. The added noise vectors have normally distributed magnitude with a standard deviation of 30nm (cf. [15, 30]) and an homogeneously distributed orientation (in 3D).

### Nonlinear FEM based TFM

**Fig 6:**
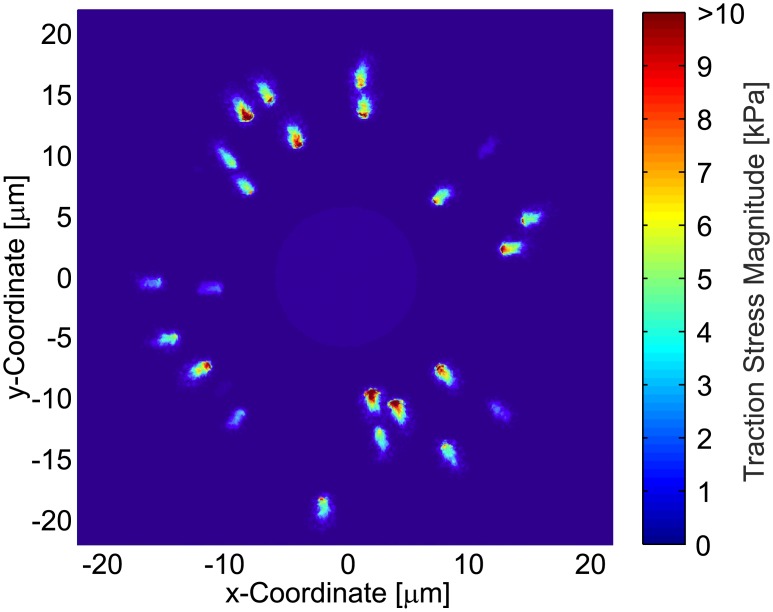
Magnitude of the reconstructed traction stress field for an in-silico generated displacement field on material A, with a displacement measurement resolution of *L*_0_ = 0.5*μm*.

The virtually measured displacement vectors are used as input data for the reconstruction of the surface traction field. To this end, a modified version of our previously developed non-linear FEM-Based TFM framework contained in the confocal TFM (cTFM) Package [30] was used. Briefly, the algorithm is composed of three principal parts: *1)* A 3D model of the substrate is created, with the same in-plane dimensions as the domain investigated in the microscopy and full substrate thickness, which is here given by the section used to create the in-silico testcase. This model is then meshed adaptively, in order to improve mesh refinement in regions with large displacements, i.e. the regions where the focal adhesions are expected to be located. *2)* The input displacement vectors, consisting of two in-plane and one out-of-plane components, are used to generate a continuous displacement field based on an interpolation scheme. In particular, either a linear triangular interpolation or a Radial Basis Function (RBF) based interpolation are applied. Compared to piecewise linear interpolation, RBF interpolation [35] not only considers data from neighbouring but also further remote data points, generally leading to a better approximation of the field between measurement positions. Additionally, the chosen thin-plate spline RBFs allow for a continuously differentiable interpolation of the displacement field. According to the interpolated displacement field, displacement boundary conditions are applied to all nodes on the top surface of the substrate which are located within the fluorescent marker array. *3)* The solution of the finite element problem is computed using an implicit non-linear FEA solver (Abaqus/Standard), taking into account the non-linear material behaviour of the substrate and the applied boundary conditions. The computed reaction forces on the top surface nodes are then related to the deformed surface areas of the connected elements to compute the reconstructed traction field (Fig 6), defined in the deformed configuration, i.e. Cauchy stress vectors.

### Quality measures for the traction force reconstruction

Similarly to the measures presented by Sabass [15], we defined quality measures for a quantitative assessment of the traction reconstruction comparing the reconstructed traction field to the reference field applied in the in-silico testcase.

**Fig 7:**
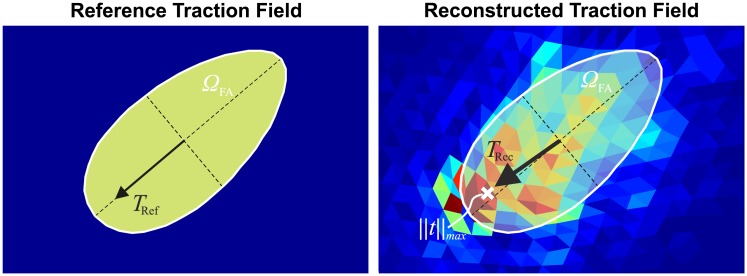
Example of the reference and reconstructed traction fields in the region of a single focal adhesion. The white contour marks the border of the FA as defined in the in-silico model. The total traction force $\vec{T}$ of the FA is plotted in both cases with a black arrow, whereas the white cross-shaped marker indicates the location with the highest traction stress within the FA.

The first measure is the Traction Magnitude Ratio (TMR)

$$\text{TMR}=\frac{\left|\right|{\vec{T}}_{\text{Rec}}\left|\right|}{\left|\right|{\vec{T}}_{\text{Ref}}\left|\right|}=\frac{\left|\right|\int_{\Omega_{\text{FA}}}{\vec{t}}_{\text{Rec}}d\Omega \left|\right|}{\Omega_{\text{FA}}\left|\right|{\vec{t}}_{\text{Ref}}\left|\right|},$$

describing the ability to reconstruct the originally applied traction force vector of the FA. The reconstructed traction force is obtained from the integral of the traction stress field over the FA surface, which is numerically evaluated by summing up the products of the polygonal areas enclosed by the FA boundary (see Fig 7) with their corresponding traction stress. Complementary to this integral measure, the Peak Traction Ratio (PTR) indicates the under- or overestimation of the maximal traction stress within the focal adhesion.

$$\text{PTR}=\frac{\text{max}(||{\vec{t}}_{\text{Rec}}\left(\Omega_{\text{FA}}\right)||)}{\left|\right|{\vec{t}}_{\text{Ref}}\left|\right|}.$$

TMR and PTR are defined locally at the level of each FA and are therefore evaluated for each of them separately, assuming that location and shape of the focal adhesions are known, which is the case for the in-silico computation. In real TFM experiments this information is typically available from the analysis of fluorescent signals of FA proteins in microscopy images (cf. [15, 22, 30, 36]).

Finally the Strain Energy Ratio (SER), a global measure of the quality of reconstruction which relates the amount of elastic work *W* of the external forces on the substrate in the reconstructed system to the work in the reference case. For a hyperelastic substrate, the strain energy is given by the volume integral of the strain energy density function defined by the constitutive equation of the material. Alternatively, for non-dissipative materials, the strain energy can be computed summing up the work of all external forces, i.e the forces which the cell induces on the substrate.

$$\text{SER}=\frac{W_{\text{Rec}}}{W_{\text{Ref}}}.$$

All proposed quality measures are defined such that their value is 1 for a perfect reconstruction, whereas values < 1 and > 1 indicate under- or overestimation, respectively.

## Results

### Displacement field resolution and interpolation

**Fig 8:**
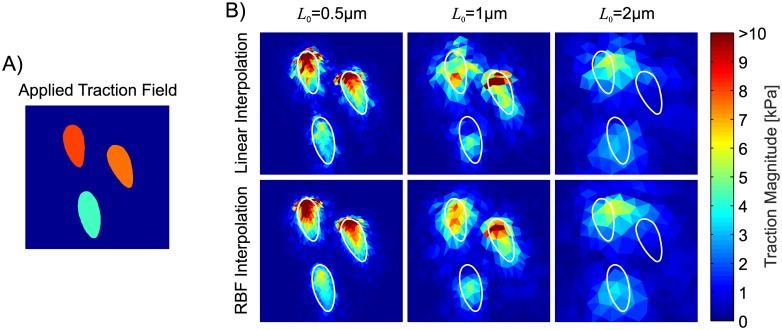
Detail of the applied (A)) and reconstructed (B)) traction field (*x* ∈ [0 6.5]*μm*, *y* ∈ [−15 −8.5]*μm*) with material A (*ν* = 0.499) for different displacement field resolutions and interpolation schemes. The white contours indicate the borders of the FAs.

Fig 8 presents the reconstructed traction stress field in the region three nearby positioned focal adhesions.

While for *L*_0_ = 0.5*μm* the reconstructed tractions are closely within the contours of the original FAs, the reconstructed traction fields become progressively blurred with increasing *L*_0_. For *L*_0_ = 2*μm*, which is equivalent to an average of 0.5 markers per FA, the reconstruction fails to resolve all three focal adhesions.

**Fig 9:**
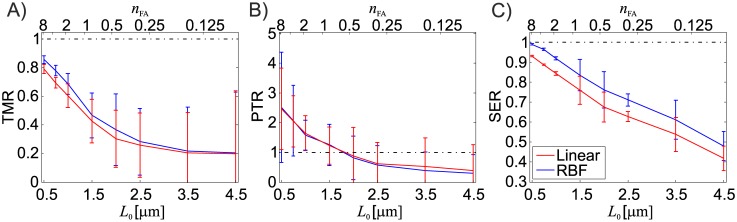
Effect of different displacement field resolutions and interpolation methods for material A (*ν* = 0.499). Reported are mean and standard deviation for 5 testcase repetitions (*N* = 125 FAs).

The qualitative observations are confirmed by the quantitative evaluations of the quality measures reported in Fig 9. Generally, the reconstructed FA traction force magnitude underestimates the applied reference force. High resolution measurements of the displacement field significantly reduces the underestimation of the traction force reconstruction (Fig 9A), but the minimum error is in the range of 15% of the reference traction force value. While high resolution also results in a more accurately captured deformation energy (Fig 9C), it is also associated with a substantial over-prediction of the reconstructed peak stresses within the FAs and large fluctuations of this value (Fig 9B). For very low resolutions (*L*_0_ > 3*μm*), the mean value of PTR and TMR stabilizes while the scatter increases with *L*_0_.

Fig 9 also underlines the influence of displacement field interpolation applied during the reconstruction, showing that both the TMR and the SER benefit significantly from a higher order interpolation scheme such as RBF, while the PTRs are found to be less affected by the interpolation method. A qualitative comparison of the reconstructed traction stress fields for the two interpolation schemes is given in Fig 8B.

### Displacement field noise

**Fig 10:**
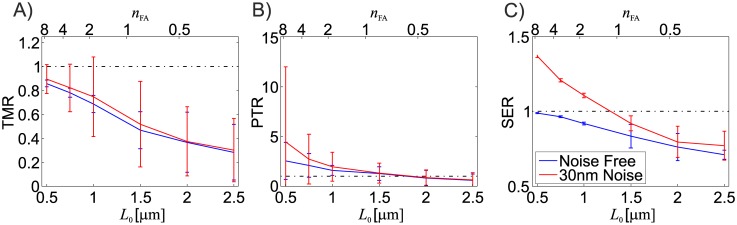
Effect of noise affected displacement vectors (Gaussian noise, 30nm standard deviation) for different displacement measurement resolutions for material A (*ν* = 0.499), solid red line. The results for the noise-free case are shown in blue. Reported are mean and standard deviation for 5 testcase repetitions (*N* = 125 FAs).

The effect of noise in the displacement field is clearly dependent on the measurement resolution itself, since for a constant noise magnitude, the relative displacement error increases with smaller *L*_0_. At high resolutions, this leads to high noise-induced traction stress peaks and to a large overestimation of the mechanical energy stored in the substrate (Fig 10C).

The results indicate that the noise leads to completely unreliable PTRs for a standard deviation of the noise amplitude above 0.02*L*_0_ (*L*_0_ ≤ 1.5*μm*, Fig 10B), the reconstructed TMR values deviate only slightly from the ones computed for the noise-free case (Fig 10A). While the latter observation is based on the mean TMR of 25 FAs, the noise induced larger variations in the results of single FAs, leading to significantly wider scatter of the TMRs for noisy traction fields indicated by large values of the standard deviation (Fig 10A).

### Out-of-plane displacement data

**Fig 11:**
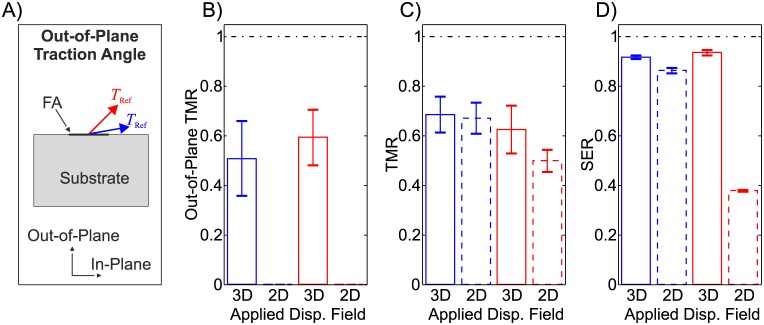
Comparison of the traction reconstruction quality measures for the reconstruction including the out-of-plane displacement field on the substrate surface (3D applied displacement field, continuous bars) and without (2D applied displacement field, dashed bars). The comparison is shown for a substrate of material A (*ν* = 0.499) and original traction fields with different out-of-plane traction angles: 10° (blue color) and 45° (red color).

The measurement of the out-of-plane plane displacement of fluorescent markers using digital image processing of microscopy images is markedly more laborious than the extraction of the associated in-plane displacement components. Importantly, for cells residing on planar substrates, the out-of-plane displacement component is usually smaller than the in-plane displacement magnitude. While it is clear that the out-of-plane displacements need to be quantified to determine the corresponding traction stress components, the effect of neglecting them in the determination of the in-plane traction field is less evident. The investigations performed on the in-silico testcase, with out-of-plane traction stress components that are significantly smaller than the in-plane components (10° out-of-plane traction angle), showed that a reconstruction performed only with the in-plane displacement data leads to results that are very close to a reconstruction accounting for the 3D surface displacement field (Fig 11C and 11D, blue bars). For this case, the main disadvantage of a reconstruction based on 2D displacement fields is therefore the loss of the ability of the TFM platform to detect traction components in the out-of-plane direction (Fig 11B).

However, the error made in the traction force magnitude by neglecting the out-of-plane displacements increases with increasing magnitude of the out-of-plane traction stresses, leading to very strong underestimation of strain energy when the out-of-plane and in-plane tractions are in the same order of magnitude. This is reflected in a significant underestimation of traction forces in during the reconstruction of such traction stress fields (45° out-of-plane traction angle, red color in Fig 11).

The comparison of the reconstruction quality of the out-of-plane traction force component and the TMR from 3D displacement measurements (continuous bars in Fig 11B and 11C, respectively) indicates that the underestimation of the out-of-plane component is larger than in the corresponding in-plane components, revealing the directional sensitivity of TFM platforms based on flat continuous substrates.

### Constitutive behaviour of the substrate material

The presented framework, together with the in-silico generation of TFM input data, enables investigating the influence of different mechanical behaviours of the TFM substrate, with the objective to identify favourable mechanical material characteristics. These observations could then be taken into account during the selection process of a substrate material for TFM experiments.

#### Deformation modes in the TFM substrate

**Fig 12:**
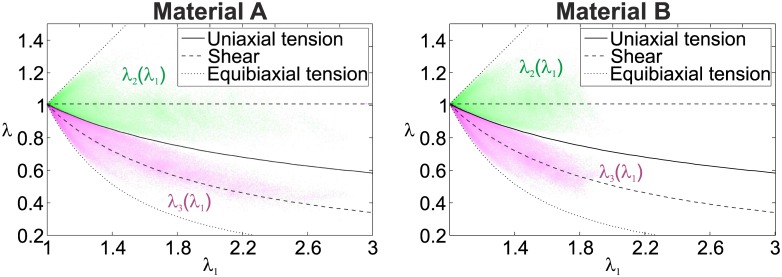
Principal Stretches *λ*_2_ (green cloud) and *λ*_3_ (violet cloud) vs. *λ*_1_ for the in-silico computation for material A and B. The color intensity is proportional to the frequency. The solid, dashed and dotted lines indicate the relations of *λ*_2_(*λ*_1_) and *λ*_3_(*λ*_1_) for the cases of uniaxial tension, pure shear and equibiaxial deformation modes, respectively.

In each finite element of the computational domain representing the substrate deformed by the traction stresses mimicking the cellular forces (in-silico testcase), the three principal stretches (*λ*_1_ > *λ*_2_ > *λ*_3_) were evaluated. Each value of *λ*_2_ and *λ*_3_ was plotted against *λ*_1_ in Fig 12. The results show that, for the proposed combination of traction magnitude and substrate stiffness, the substrate material is effectively subjected to very large strains. Additionally, independent of the material type, the highly stretched substrate regions are mostly subjected to a (pure) shear deformation mode, indicated by a clustering of the principal stretch data around the *λ*_2_ = 1 and $\lambda_{3}=\lambda_{1}^{-1}$ lines. The non-linearity of the stress-strain relationship of the material influences the magnitude of the deformations, here shown by the lower magnitude of the principal stretches exhibited in material B.

#### Nonlinear elastic behaviour

**Fig 13:**
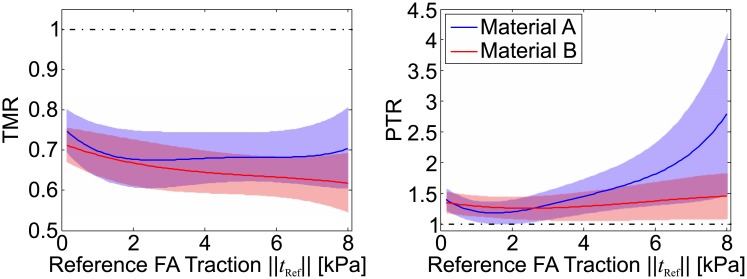
Reconstruction quality measures against nominal focal adhesion traction stress *t*_*Real*_ for material A and B with *ν* = 0.499. Reported is mean (solid line) and standard deviation (shaded area) for N = 25.

All the 25 FAs in the testcase pull with different reference traction values, allowing to report the results of the quality scores as a function of the reference traction value to assess the effect of geometrical and material non-linearity. In general terms, the accuracy of the reconstruction decreases slightly with increasing reference FA traction stresses. The results reported in Fig 13 underline that the use of a material with a strain stiffening behaviour (such as material B) increases the quality of the reconstruction by substantially reducing the spurious traction stress peaks for focal adhesions exerting high forces. At the same time, the errors in the traction forces (Fig 13) of the reconstruction based on material B remains in the same range as for Material A.

#### Volumetric behaviour

Since TFM based on 3D displacement data leads to a severely constrained mechanical problem, it is expected that the volumetric behaviour of the substrate material strongly affects the reconstruction, particularly in combination with noisy displacement fields.

**Fig 14:**
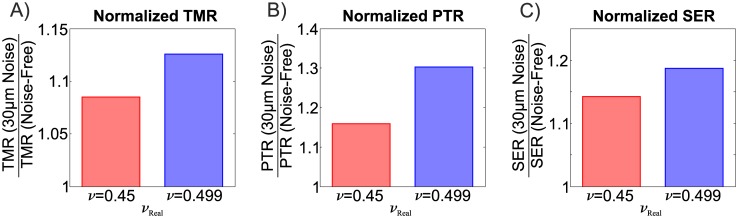
Influence of noise on the reconstruction for compressible (*ν* = 0.45, red bars) and nearly incompressible (*ν* = 0.499, blue bars) substrates. The results are presented normalized to the respective noise free cases to highlight the relative change induced by the noise. Note that the same volumetric behaviour (*ν*_*Rec*_ = *ν*_*Real*_) was assumed for generating the test case and its reconstruction.

Fig 14 reports the influence of noise on the traction reconstruction quality measures for increasing approximation to incompressible substrate behaviour (*ν* = 0.45 and *ν* = 0.499). The results indicate that slightly compressible substrate materials offer a substantial advantage regarding the sensitivity to noise. When subjected to noise with equal magnitude, the less compressible substrate material (*ν* = 0.499) leads to significantly higher deviations from the noise-free traction reconstruction.

### Modelling of geometric and material nonlinearities

The large strain deformations and the complex material behaviour have to be taken into account for a correct representation of the physical processes occurring during TFM. In this section, the errors induced by a linearisation of the large-strain TFM problem are quantified. To this end, for the solution of the inverse problem, linear material behaviour and the linear solver of the FEA software were used, thus neglecting both, material and geometric nonlinearities induced by large deformation.

**Fig 15:**
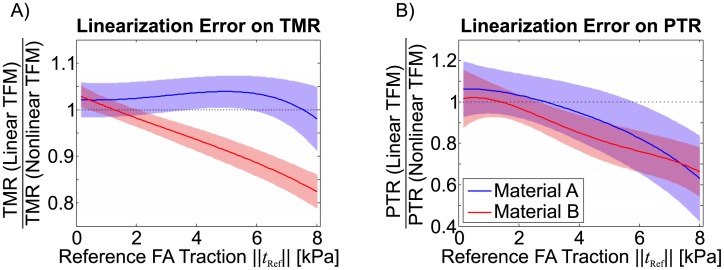
Comparison of the reconstructed traction force (TMR) and traction stress peaks (PTR) of single FAs with different traction magnitude for the linear TFM and the fully non-linear TFM for materials A and B. Reported is mean (solid line) and standard deviation (shaded area) for N = 25.

The comparison of the reconstruction quality based on either the non-linear and the linear FEA (Fig 15A) shows that for low FA traction stress magnitudes (≤3 kPa) both analyses yield similar results. For material B, with nonlinear stress-strain characteristics, the results of the linear FEA increasingly differ from the non-linear one at higher traction stresses. Material A, on the contrary, leads to similar results even for large traction values. Interestingly, the linearised reconstruction has a similar influence on the PTR for both materials, (Fig 15B) underestimating up to 30% the traction peaks compared to the nonlinear analysis.

## Discussion

**Fig 16:**
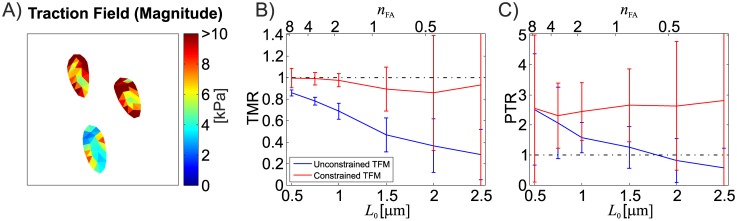
A) Traction stress field reconstruction (resolution *L*_0_ = 1*μm*) achieved by additionally accounting for FA position and shape (constrained method). The same region as reported in Fig 8 is shown. B) and C) Comparison of the performance of the unconstrained (blue) and constrained (red) reconstruction scheme for the noise free case and Material A. Reported are mean and standard deviation for 5 testcase repetitions (*N* = 125 FAs).

Due to the nature of the inverse problem of TFM, for a finite resolution, the (noise free) reconstruction always underestimates the mechanical energy in the TFM substrate (Fig 9C). This is due to the finite distance between fluorescent markers which defines the minimal wavelength of perturbations in the displacement field that can be resolved and therefore be taken into account during the traction reconstruction. Since the reconstruction is a displacement controlled mechanical problem, this underestimation of strain energy is translated into an underestimation of the traction forces induced into the substrate by the cell’s focal adhesions (Fig 9A). The underestimation of FA traction forces for the analysed resolutions using nonlinear TFM is comparable to underestimations reported by Sabass for the linear TFM algorithm FTTC [11] using a slightly different testcase [15]. Besides the underestimation of strain energy, the underestimation of FA traction forces is additionally aggravated by the inability to reproduce the discrete contours of the FA due to the finite resolution, leading to a spatial blurring of its traction stresses. Fractions of the reconstructed traction stresses are therefore erroneously associated with areas outside the FA area (Fig 8) and not taken into account for the integral FA traction force. This is an intrinsic limitation of unconstrained TFM methods, which can only be solved taking into account additional information, In particular, information on FA location and shape available from fluorescent microscopy imaging (cf. [15, 22, 30, 36]) can be used to constrain the reconstructed traction field to be zero outside the FAs. An example of such a constrained TFM implementation is the Boundary Element Method for linear TFM proposed by Dembo [10]. In fact, constraining the reconstructed traction stress field in our large strain TFM method led to a significant improvement of performance in terms of traction force reconstruction and it allowed to almost eliminate the force underestimation for resolutions up to *L*_0_ = 1.5*μm* (Fig 16B). However, the constrained reconstruction also tends to an overestimation of traction peaks (Fig 16C). It should be mentioned, that the location of the FAs is measured in the deformed configuration, while the large deformation TFM problem is formulated in the reference configuration. This requires the FA contours to be mapped back into the reference configuration, which is expected to result in an increased displacement field noise sensitivity for constrained large deformation TFM methods. It is important to note that while the present study assumes that the location and shape of each FA is exactly known, the experimental uncertainty on FA localization is expected to induce corresponding errors in the evaluation of FA traction forces for both, the constrained and the unconstrained method.

Compared to the underestimation of FA traction forces due to the finite resolution, which is in the order of 25% for a marker spacing of 1*μm*, some of the analysed factors have only little influence on the reconstruction. While applying a linear interpolation scheme used for the displacement field instead of RBF interpolation results in an additional underestimation of the traction forces by about 10%, neglecting the out-of-plane displacement field component results only in a marginal deterioration of TFM reconstruction when cells are mostly pulling in the in-plane directions. While all the presented reconstruction results were obtained from TFM computations with a substrate model that reproduced the thickness of the reference case, additional calculations were performed to quantify the reconstruction error in case of an assumption of a half-space substrate (such as for classical linear TFM implementations). The associated relative error on TMR and PTR was -5% and -10% respectively. Significantly larger errors, in the range of 10% for TMR and 39% for PTR, were observed for a case in which the thickness of the substrate used for the reconstruction was lower than the real one (20μm instead of 30μm). Note however that the influence of substrate thickness is related to the size of the cell.

**Fig 17:**
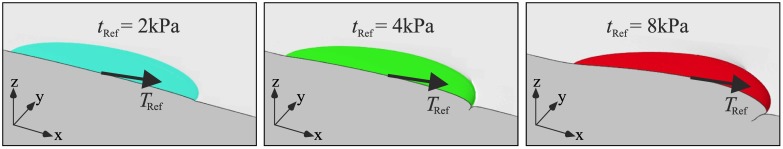
Cross-section of the substrate (dark grey, *xz*-plane) underneath the focal adhesion (coloured half-ellipse), highlighting the deformation and surface wrinkling under increasing FA traction *t*_Ref_.

The performed studies highlight that the measurement resolution of the displacement field in TFM, defined by the density of measurement points on the substrate-cell interface is the key factor for high quality TFM analyses together with a reliable resolution of the focal adhesion attachment sites (cf. [15]). However, the results also highlight that while the reconstructed traction forces and strain energy converge to the reference values for a higher resolution, the peak tractions within the focal adhesions do not follow this trend and are significantly overestimated (Fig 9B). The results presented in Fig 13 indicate that the magnitude of overestimation of traction peaks depends on the reference FA traction force magnitude, increasing strongly for FAs pulling with high force, and can be significantly reduced by using a material with a progressive (i.e nonlinear stiffening) stress-strain response. This suggests that the issue is related to the high deformations associated with large FA tractions. A more detailed analysis of the reference deformation pattern in the region of a single FA (reported in Fig 17) reveals that the increasing surface traction induced by the FA causes the substrate to wrinkle at the leading edge, due to the compression of the substrate. Such a localized deformation field cannot be reproduced by the interpolation scheme for the displacements, thus leading to a localized overestimation of the stiffness at the leading edge. The divergence of the reconstructed traction peaks at high resolution reported in Fig 9B as the average of all FAs is therefore driven by the FAs associated with such large deformations. Further, the centre point of the traction stress distribution over the FA tends to move towards the leading edge (see Fig 8), indicating that the analysis of the stress field distribution within the FA might be significantly affected by reconstruction artefacts.

This phenomenon is expected to affect the reconstruction of the traction magnitude within each FA in case of large deformations, thus even application of a fully non-linear FEA based TFM algorithm is not sufficient to avoid localization effects occurring in very soft substrates. From a mechanical perspective, the substrate stiffness should be selected depending on the magnitude of traction forces exerted by the analysed cell in order to avoid excessive surface wrinkling. The non-linear analysis offers the advantage of handling correctly material non-linearities, allowing the use of TFM substrates which are soft in the low-strain regime and stiffen with increasing deformation. Such material non-linearity allows to combine the high traction force detecting sensitivity of soft substrates with the benefit of significantly reduced deformations around strong FAs, thus extending the range of detectable traction magnitudes. As a result, the reconstruction quality can be maintained in terms of both traction forces as traction peaks over a large range of traction magnitudes. It is important to note that this also implies the use of a nonlinear solver, since the linearised solution leads to increasing reconstruction errors for large FA forces (Fig 15). The latter results on the influence of geometrical and material nonlinearities have been found to be in good qualitative agreement with previous studies [19, 20]. A combination of the results presented in Figs 15 and 4B reveals that the maximal displacement magnitude that could be treated by means of a linearised solution method is about 600nm and 300nm for the fairly linear material A and the nonlinear material B, respectively.

Another material characteristic which has been proven advantageous for TFM is compressibility. For noise-affected displacement fields, a (slightly) compressible substrate performs better than an almost incompressible material due to the lower strain energy contribution associated with noise induced volumetric deformations. In TFM computations, noise leads to an overestimation of mechanical energy in the substrate and increases additionally the overestimation of traction peaks (Fig 10). Under the assumption that the magnitude of the displacement field noise is independent of the measurement resolution, the influence of noise on the quality of the reconstruction increases strongly with the resolution, since the noise induced fluctuations have shorter wavelength at high resolutions, resulting in higher local deformation gradients. Noise at high resolutions has therefore to be handled with regularization schemes, such as L1 or L2 regularization [23, 24] if traction stress peaks need to be resolved correctly. Interestingly, the total reconstructed traction force exerted by the focal adhesions is markedly less noise-sensitive, enhancing the conclusion that the FAs traction force, defined as an integral measure, is a much more reliable indicator of the mechanical interaction of the cell with the substrate than traction stress peaks.

## Conclusions

This study investigated in detail the mechanical analysis associated with TFM, allowing to assess the importance and quantify the influence of several factors. We found that the errors in quantitative TFM results can be significant and strongly depend on the displacement field resolution, i.e. on the quantity of measurement locations within each FA. High resolution of the measured displacement field increases the quality of the detected tractions integrated over the FA (i.e. the traction force), but strongly aggravates the misestimation of traction peaks within an FA in presence of noise as well as without noise in the case of larger substrate deformation. The reconstruction errors of the FA force and peak traction stress, inherent to TFM analyses have been found to be dependent on both the resolution and the magnitude of traction stress, requiring a careful assessment of TFM results when using these metrics to quantify the mechanical interaction of a cell with a soft substrate. We found that a linearised treatment of the TFM problem performs reasonably well for quasi-linear substrates in terms of traction force reconstruction also for deformations beyond the linear regime. However, the use of non-incompressible and non-linear stiffening substrates provides more reliable results and allows a higher quality in reconstruction over a wider range of FA forces, but requires a nonlinear TFM solution algorithm.

In conclusion, experimental TFM setups and reconstruction algorithms need to be selected with respect to the expected cell forces and substrate deformations. In this regard, platforms using both nonlinear substrates and analyses bring an advantage in terms of the range of applicability and reliability of TFM.
